# Integrating Single-Cell, Bulk, and Spatial Transcriptomics Unveils a Novel Ribosome Biogenesis-Related Prognostic Model and Defines *RPS19BP1* as a Pro-Oncogenic Regulator in Lung Adenocarcinoma

**DOI:** 10.3390/ijms27135864

**Published:** 2026-06-29

**Authors:** Shengze Chen, Pengfei Du, Qiang Luo, Shuang You, Dingkun Huang, Qian Ou, Mingyi Zhang, Leichong Chen, Dejun Zhang, Rui Meng

**Affiliations:** 1 First Clinical College, Union Hospital, Tongji Medical College, Huazhong University of Science and Technology, Wuhan 430022, China; chenshengzesh@163.com (S.C.); m202576265@hust.edu.cn (P.D.); lqwalter@163.com (Q.L.); isysysuu@163.com (S.Y.); huangdk17@163.com (D.H.); ouqianooo@163.com (Q.O.); shahuhu6789@gmail.com (M.Z.); clc99806@163.com (L.C.); zhangdejun@hust.edu.cn (D.Z.); 2 Cancer Center, Hubei Key Laboratory of Precision Radiation Oncology, Institute of Radiation Oncology, Union Hospital, Tongji Medical College, Huazhong University of Science and Technology, Wuhan 430022, China; 3 Key Laboratory of Biological Targeted Therapy, Huazhong University of Science and Technology, Ministry of Education, Wuhan 430022, China

**Keywords:** lung adenocarcinoma, ribosome biogenesis, prognosis, *RPS19BP1*, tumor immune microenvironment

## Abstract

Dysregulation of ribosome biogenesis is increasingly recognized as a hallmark of tumor malignancy, yet its prognostic implications in lung adenocarcinoma (LUAD) remain incompletely characterized. This study aimed to construct a ribosome biogenesis-related prognostic model for LUAD and explore its potential relevance to the tumor immune microenvironment. Single-cell and bulk RNA sequencing data were integrated to identify ribosome biogenesis-related genes (RBRGs), from which a prognostic risk score was established via Cox regression, LASSO regression, and multivariate Cox analyses and validated in two independent GEO cohorts. Associations between the risk score and tumor mutation burden, immune infiltration, and computationally inferred immunotherapy response were systematically evaluated. In vitro experiments were performed to characterize the biological function of *RPS19BP1*, a key gene in the model. A total of 262 RBRGs were identified, and the derived 14-gene risk score demonstrated prognostic value across three cohorts (TCGA: 1-, 2-, 3-year AUC = 73.08, 72.44, 72.20; GSE68571: 1-, 2-, 3-year AUC = 67.93, 73.24, 77.59; GSE8894: 1-, 2-, 3-year AUC = 75.56, 72.99, 71.77). The low-risk group exhibited a more immunocompetent tumor microenvironment, whereas the high-risk group was associated with an immunosuppressive phenotype. Knockdown of *RPS19BP1* significantly attenuated the proliferation, migration, and invasion of LUAD cells. This multi-omics-derived prognostic model showed prognostic potential in retrospective LUAD cohorts, is associated with distinct immune infiltration patterns, and identifies *RPS19BP1* as a pro-oncogenic regulator in LUAD.

## 1. Introduction

Lung cancer is the most common cancer worldwide, with approximately 2.5 million new cases and 1.8 million cancer-related deaths in 2022, imposing a substantial societal burden and economic loss [1]. Among all lung cancer subtypes, non-small cell lung cancer (NSCLC) accounts for 80–85% of cases, representing the most prevalent histological type. Within NSCLC, lung adenocarcinoma (LUAD) and lung squamous cell carcinoma (LUSC) are the most common subtypes [2]. Although conventional therapies, including radiotherapy, chemotherapy, and surgical resection, have been well established, their efficacy is limited by poor bioavailability, high drug resistance rates, and a high incidence of tumor recurrence, particularly in patients with advanced LUAD [3]. In recent years, advances in understanding the heterogeneity of distinct molecular subtypes of LUAD and the plasticity of the tumor microenvironment (TME) [4,5] have facilitated the development of immune checkpoint blockade (ICB) therapy, a personalized therapeutic approach that has improved the prognosis of LUAD patients to some extent [6]. Nevertheless, not all LUAD patients derive clinical benefit from immunotherapy. Therefore, accurately predicting the prognosis of LUAD patients and precisely identifying those most likely to benefit from immunotherapy have emerged as pressing clinical challenges that remain to be addressed.

Ribosome biogenesis is a precisely orchestrated and highly complex biological process [7,8,9], encompassing rRNA transcription, processing, and modification, as well as ribosome assembly and pre-ribosome export [10]. This process involves a large repertoire of assembly and maturation factors, and its activity is governed by diverse cellular signaling pathways [11]. In recent years, a growing body of evidence has indicated that increased ribosome abundance and altered ribosomal modifications are closely associated with the initiation and progression of multiple cancers [12,13]. Wang et al. demonstrated that SOD1 promotes the progression of *KRAS*-driven NSCLC by upregulating ribosome biogenesis [14]. Pan et al. reported that ribosome biogenesis factors (RBIS) are intimately involved in LUAD progression and represent a potential therapeutic target [15]. Nevertheless, the impact of ribosome biogenesis on the proliferation, invasion, and immune infiltration of LUAD has yet to be fully elucidated.

With the advancement of molecular biology techniques, scRNA-seq has overcome the inherent limitations of conventional bulk RNA-seq approaches. By enabling transcriptomic profiling at single-cell resolution, scRNA-seq allows the dissection of cellular heterogeneity within diverse tissues and facilitates the precise characterization of distinct cell subpopulations [15,16]. In the field of oncology, scRNA-seq has emerged as a pivotal technology for investigating the tumor microenvironment (TME), identifying novel molecular subtypes, uncovering potential therapeutic targets, and defining prognostic biomarkers. Complementary to scRNA-seq, spatial profiling technologies enable the measurement of transcript abundance within intact tissue sections while preserving authentic spatial coordinates, thereby providing an intuitive visualization of gene expression patterns and the physical distribution of cell populations [17]. In cancer research, this approach has become an essential tool for interrogating intercellular communication among cell subpopulations and evaluating the extent of tumor immune infiltration. Therefore, the integration of scRNA-seq and spatial profiling technologies holds considerable promise for identifying key biomarkers in LUAD and characterizing their expression landscapes, which may ultimately contribute to predicting therapeutic response, patient prognosis, and disease progression.

In this study, we leveraged scRNA-seq data to identify distinct cell types within LUAD specimens and systematically characterized the ribosome biogenesis activity levels across these cell populations. Building on these findings, we integrated bulk RNA-seq data to construct a risk score and prognostic model capable of accurately predicting the prognosis of LUAD patients, and explored its potential relevance to immunotherapy response. Importantly, we performed an in-depth analysis of the key gene *RPS19BP1*, focusing on its potential biological functions in lung cancer cells. For the first time, we experimentally validated the oncogenic role of *RPS19BP1* in LUAD through in vitro functional assays.

## 2. Results

### 2.1. Single-Cell Transcriptomic Analysis

**Figure 1:**
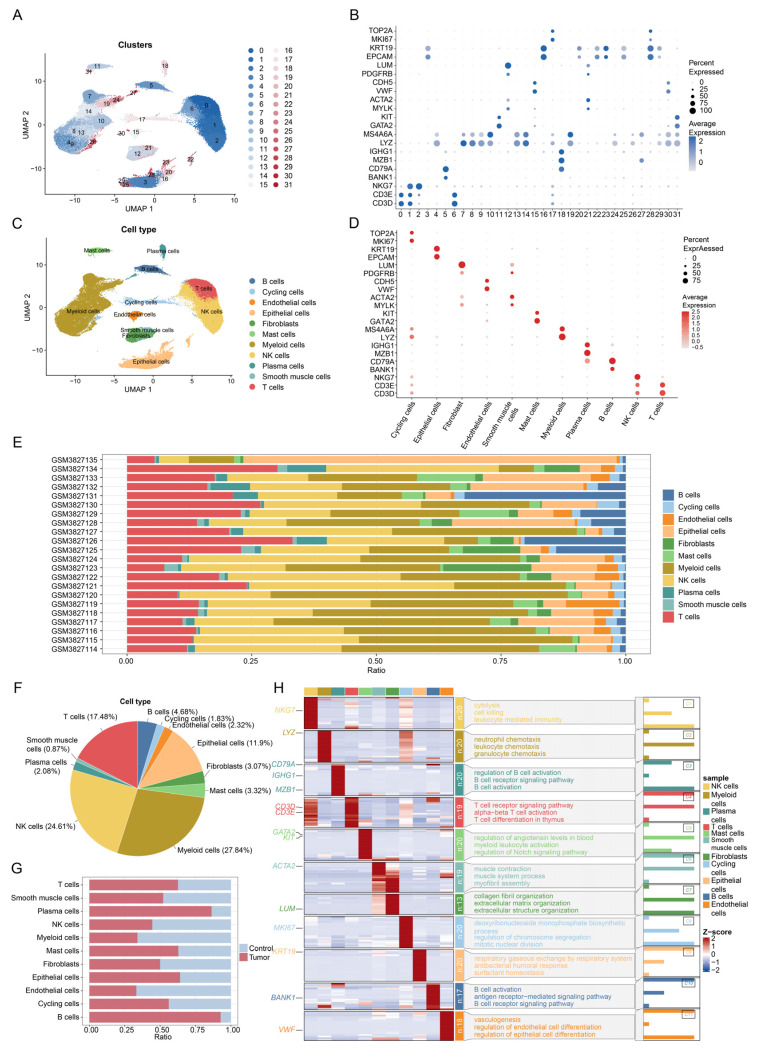
scRNA-seq data analysis for screening ribosome biogenesis-related genes. (**A**) Principal Component Analysis (PCA) and Uniform Manifold Approximation and Projection (UMAP) clustering divided cells into 32 clusters. (**B**) Bubble plot showing the major differentially expressed markers across different clusters. (**C**) Clusters were classified into 11 distinct cell types by manual annotation, and the markers for different cell types are presented as a bubble plot in (**D**). (**E**) Cell type proportions for each sample. The overall cell type proportions and the cell type proportions for normal and tumor tissues are shown in (**F**) and (**G**), respectively. (**H**) Heatmap shows the top three enriched biological process (BP) terms from GO enrichment analysis for each cell type.

We obtained 11 paired lung cancer and adjacent normal lung tissue samples (GSE131907) from the Gene Expression Omnibus (GEO) database. Following stringent quality control procedures, a total of 86,556 high-quality cells were retained for downstream analysis. Using principal component analysis (PCA) and UMAP dimensionality reduction, the cells were partitioned into 32 subclusters (Figure 1A). Concurrently, marker genes exhibiting high heterogeneity across cell subclusters were visualized using a bubble plot (Figure 1B). Subsequently, comprehensive cell annotation was performed. The results demonstrated that these cells were precisely classified into 11 distinct cell types, including epithelial cells, mesothelial cells, fibroblasts, mast cells, myeloid cells, NK cells, plasma cells, B cells, smooth muscle cells, T cells, and cycling cells (Figure 1C). To intuitively illustrate the key differentially expressed genes across cell types, a bubble plot was employed for visualization (Figure 1D). The proportional composition of each cell type across individual samples and different groups was depicted using stacked bar plots (Figure 1E,G), while the overall cell-type proportions were represented as a pie chart (Figure 1F). To validate the accuracy of our manual annotation, we performed Gene Ontology (GO) enrichment analysis on the key differentially expressed genes of each cell type and presented the top three enriched biological process (BP) terms (Figure 1H).

### 2.2. Identification of Ribosome Biogenesis-Related Genes (RBRGs)

**Figure 2:**
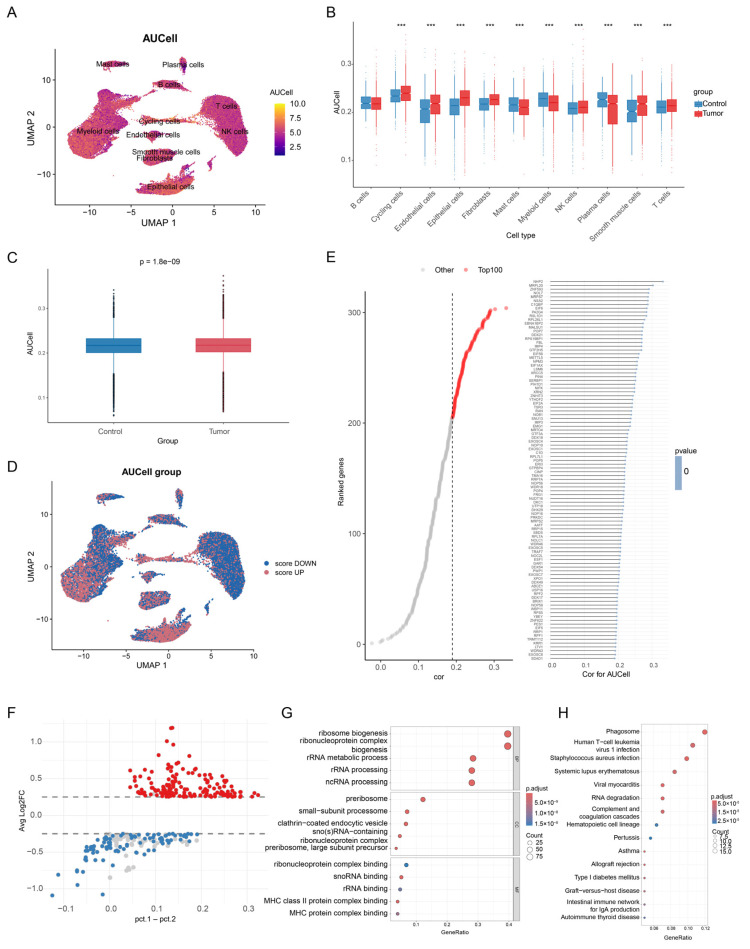
AUCell score of ribosome biogenesis in different cells and screening of Ribosome Biogenesis-related Genes (RBRGs). (**A**) The AUCell score represents the abundance of ribosome biogenesis in each cell, which is intuitively visualized by a gradient color scheme. (**B**) The comparison results of AUCell scores of various cell types between the normal group and tumor group are presented in a boxplot. (**C**) Overall AUCell scores of the normal group and tumor group. (**D**) Cells in the tumor group were stratified into distinct subgroups based on the median AUCell score. The red color denotes the subgroup with a high level of ribosome biogenesis, while the blue color indicates the subgroup with a low level of ribosome biogenesis. (**E**) Correlation analysis between gene expression levels and AUCell scores. The top 100 genes with the highest correlation are displayed in a gradient manner ranked by *p* value. (**F**) The volcano plot illustrates the differentially expressed genes between the high and low AUCell score groups. (**G**,**H**) The Gene Ontology (GO) and Kyoto Encyclopedia of Genes and Genomes (KEGG) enrichment analysis results of RBRGs are presented in the form of bubble plots. (*** means *p* < 0.001).

To investigate the ribosome biogenesis activity levels across different cell types in LUAD tissues and adjacent normal lung tissues, we employed the AUCell algorithm to evaluate the ribosome biogenesis activity of each individual cell (Figure 2A), with cells exhibiting higher expression of signature genes displaying elevated AUC scores. The results revealed that both the overall AUC score of LUAD tissue samples and the AUC scores of the majority of cell types were higher than those of normal lung tissue samples, suggesting an upregulation of ribosome biogenesis activity in LUAD tissues (Figure 2B,C). Subsequently, we stratified cells in the LUAD group into an AUCell score-high group and an AUCell score-low group using the median AUC score as the threshold. The results indicated that cells in the AUCell score-high group were predominantly enriched in myeloid cells and epithelial cells (Figure 2D). We then extracted the top 100 genes most strongly correlated with the AUC score and performed differential expression gene (DEG) analysis (adjusted *p* value < 0.05) between the AUCell score-high and AUCell score-low groups, yielding a total of 319 differentially expressed genes, of which 165 genes were upregulated (log2 fold change(FC) > 0) and 154 genes were downregulated (log2 fold change(FC) < 0) (Figure 2E,F). By taking the union of the top 100 AUC-correlated genes with the 165 upregulated DEGs (Tables S1 and S2), we ultimately obtained 262 ribosome biogenesis-related genes (RBRGs). A Venn diagram illustrated the specific composition of the 262 RBRGs (Figure S1A). KEGG and GO enrichment analyses of the RBRGs revealed significant enrichment of ribosome processing-related pathways (Figure 2G,H).

### 2.3. Construction and Validation of a Prognostic Risk Score Model Based on RBRGs

**Figure 3:**
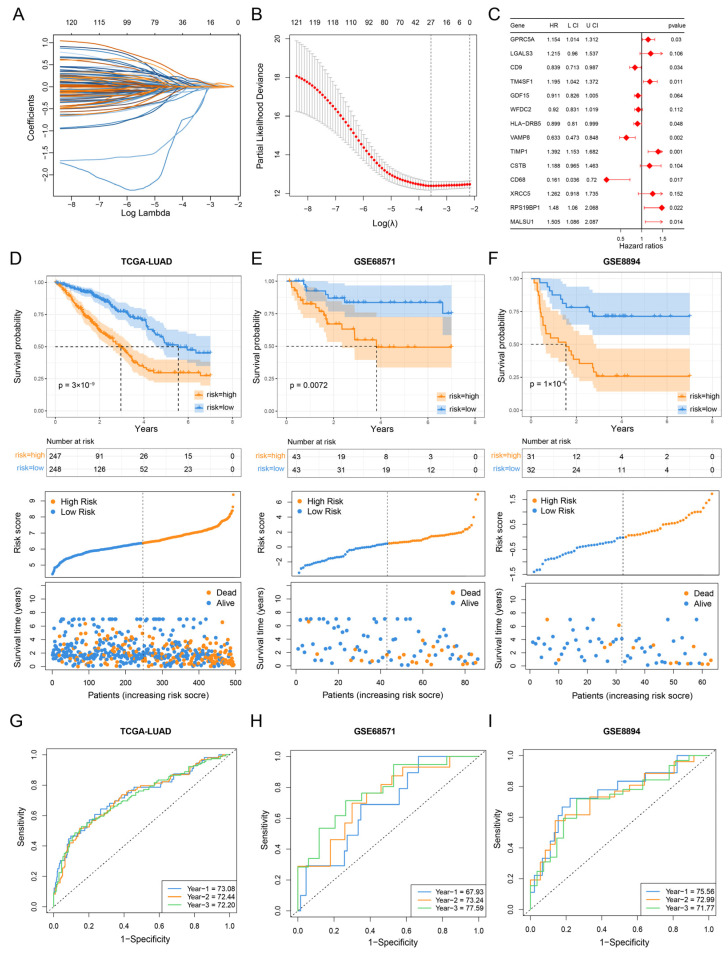
Construction of a prognostic model based on RBRGs. (**A**,**B**) A total of 27 genes with non-zero Least Absolute Shrinkage nd Selection Operator (LASSO) regression coefficients were identified via LASSO regression analysis. (**C**) The results of multivariate Cox regression analysis are presented in the form of a forest plot. (**D**) Kaplan–Meier curves of overall survival (OS) based on the risk signature in the TCGA dataset and the distribution of patient risk scores. (**E**,**F**) Kaplan–Meier curves of OS based on the risk signature in the GEO datasets (GSE68571, GSE8894). (**G**) Time-dependent ROC curves of the risk signature based on the TCGA dataset. (**H**,**I**) Time-dependent ROC curves of the risk signature based on the GEO datasets (GSE68571, GSE8894).

Of the 262 RBRGs identified, 7 were not detected in the TCGA-LUAD expression matrix and were excluded from downstream analyses, resulting in a final set of 255 genes. To further identify RBRGs associated with patient prognosis, we extracted the detected 255 RBRGs from the TCGA-LUAD cohort and performed univariate Cox regression analysis, which preliminarily identified 124 candidate genes significantly associated with prognosis. To further refine the core prognostic RBRGs, we employed LASSO regression analysis, which identified 27 genes with non-zero LASSO coefficients, including *FBP1*, *GPRC5A*, *KRT8*, *LGALS3*, *PKM*, *SFTA3*, *SLC34A2*, *ERRFI1*, *CD9*, *TM4SF1*, *TXN*, *GDF15*, *WFDC2*, *KRT7*, *HLA-DRB5*, *SFTPB*, *VAMP8*, *TIMP1*, *COL1A1*, *CSTB*, *CD68*, *HLA-DQA1*, *WDR18*, *TMA16*, *XRCC5*, *RPS19BP1*, and *MALSU1* (Figure 3A,B). Finally, multivariate Cox regression analysis was applied to determine the final 14 core prognosis-associated RBRGs and to estimate the model coefficients (Figure 3C). The risk score model was calculated according to the following formula: riskscore = 0.143 × *GPRC5A* + 0.195 × *LGALS3* − 0.175 × *CD9* + 0.179 × *TM4SF1* − 0.093 × *GDF15* − 0.083 × *WFDC2* − 0.106 × “*HLA-DRB5*” − 0.457 × *VAMP8* + 0.331 × *TIMP1* + 0.172 × *CSTB* − 1.827 × *CD68* + 0.233 × *XRCC5* + 0.392 × *RPS19BP1* + 0.409 × *MALSU1*. The TCGA-LUAD cohort was designated as the training set, while two independent external cohorts, GSE68571 and GSE8894, were employed as validation sets. Patients were stratified into high-risk and low-risk groups using the median risk score as the threshold to assess the risk stratification capacity of the model. Kaplan–Meier survival analysis revealed significant differences in overall survival (OS) between the two risk groups across the TCGA-LUAD cohort (*p* = 3 × 10^−9^), the GSE68571 cohort (*p* = 0.0072), and the GSE8894 cohort (*p* = 1 × 10^−4^), with patients in the high-risk group consistently exhibiting shorter OS (Figure 3D–F). Furthermore, time-dependent receiver operating characteristic (timeROC) analysis was performed to evaluate the predictive accuracy of the prognostic model. In the TCGA-LUAD training set, the area under the curve (AUC) values at 1, 2, and 3 years reached 73.08, 72.44, and 72.20, respectively; in the GSE68571 validation set, the AUC values at 1, 2, and 3 years were 67.93, 73.24, and 77.59, respectively; and in the GSE8894 validation set, the AUC values at 1, 2, and 3 years were 75.56, 72.99, and 71.77, respectively (Figure 3G–I). The prognostic model demonstrated discriminative performance across all three cohorts, with C-index values of 0.699 (95% CI: 0.653–0.745) in the TCGA-LUAD training cohort, 0.705 (95% CI: 0.610–0.800) in the GSE68571 validation cohort, and 0.689 (95% CI: 0.591–0.787) in the GSE8894 validation cohort. In addition, the 1, 2, and 3 year calibration curves for the risk score demonstrated favorable predictive accuracy of the model (Figure S2A). Univariate and multivariate Cox regression analyses incorporating the risk score alongside conventional clinical factors (including age, sex, and TNM stage) demonstrated that the risk score retained independent prognostic value (Figure S2B,C). Finally, a nomogram integrating T stage, N stage, and the riskscore was constructed to predict the 1, 2, and 3 year OS probabilities of LUAD patients (Figure S2D). Calibration curves (Figure S2E) and decision curve analysis (DCA) (Figure S2F) indicated that the nomogram has potential utility in predicting the prognosis of LUAD patients. Collectively, these results indicate that the prognostic model represents a reliable tool for predicting the prognosis of LUAD.

### 2.4. Mutational Landscape and Immune Characterization of the Risk Score Prognostic Model

**Figure 4:**
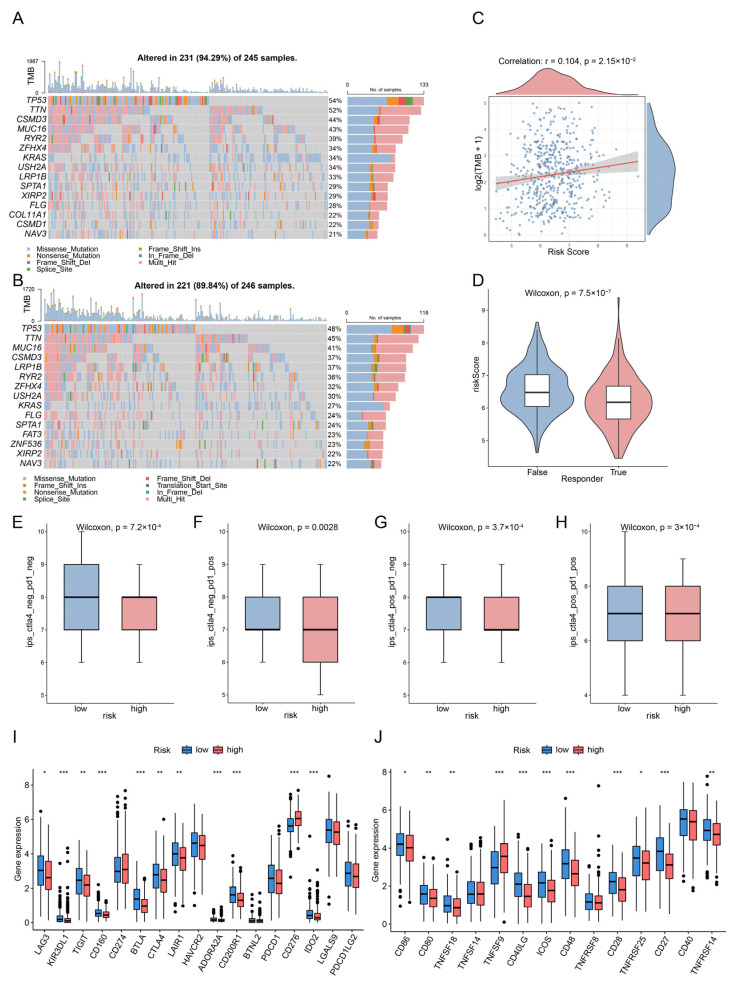
Tumor mutational burden (TMB) analysis and immune-related analysis of different risk groups. (**A**) The TMB status in the high-risk group visualized via a waterfall plot. (**B**) The TMB status in the low-risk group visualized via a waterfall plot. (**C**) Correlation analysis between TMB and risk score. (**D**) The violin plot shows a comparison of the risk scores among patients with different immune therapy responses. (**E**–**H**) The comparison of IPS between the high- and low-risk groups visualized using boxplots. (**I**,**J**) The comparison of immunosuppressive and immune-activated checkpoints between the high- and low-risk groups visualized using boxplots. (* means *p* < 0.05; ** means *p* < 0.01; *** means *p* < 0.001).

**Figure 5:**
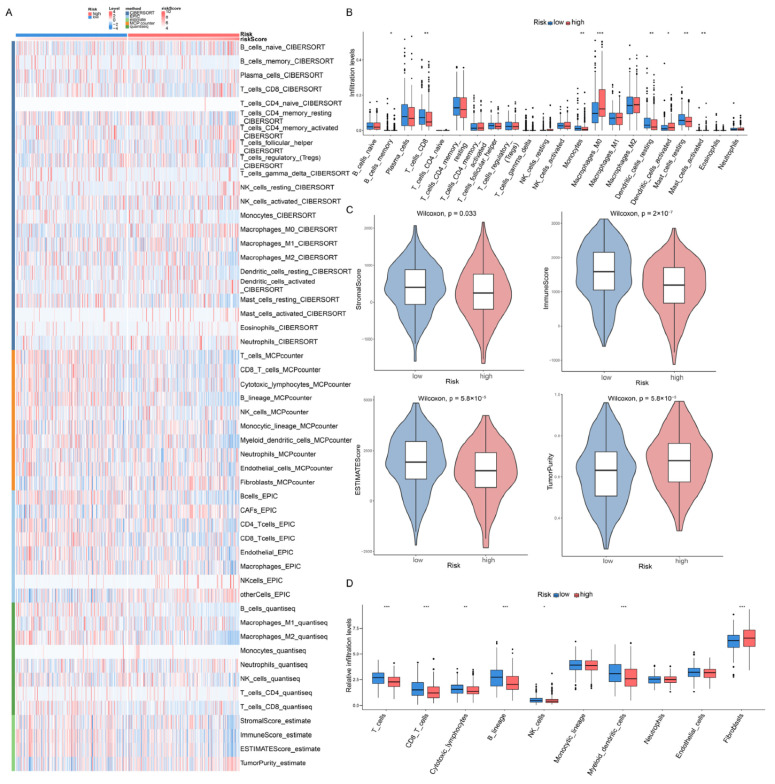
Immune infiltration characteristics of high-risk and low-risk groups. (**A**) The heatmap shows the infiltration levels of different immune cells in the high-risk and low-risk groups calculated by five different algorithms. (**B**) The comparison of CIBERSORT analysis results between the high-risk and low-risk groups visualized in the form of a boxplot. (**C**) The comparison of ESTIMATE analysis results between the high- and low-risk groups visualized by violin plots. (**D**) The comparison of MCP-counter analysis results between the high-risk and low-risk groups visualized in the form of a boxplot. (* means *p* < 0.05; ** means *p* < 0.01; *** means *p* < 0.001).

To further investigate the mutational burden across different risk groups, we visualized the oncoplot of somatically mutated genes in the high-risk group (Figure 4A) and the low-risk group (Figure 4B) of the TCGA-LUAD cohort. The results demonstrated that the mutation frequencies of *TP53*, *TTN*, *CSMD3*, *MUC16*, *RYR2*, *ZFHX4*, *KRAS*, *USH2A*, *LRP1B*, *SPTA1*, *XIRP2*, and *FLG* were consistently higher in the high-risk group than in the low-risk group, indicating an elevated overall mutational burden. In addition, the risk score exhibited a positive correlation with tumor mutational burden (TMB) (Figure 4C). Tumor Immune Dysfunction and Exclusion (TIDE) analysis revealed that patients inferred as non-responders by the TIDE algorithm displayed significantly higher risk scores (*p* = 7.5 × 10^−7^) (Figure 4D). Moreover, patients in the low-risk group consistently exhibited higher IPS across all four immune status categories compared with those in the high-risk group, suggesting that the low-risk group may possess stronger overall immunogenicity, which could be associated with a greater likelihood of benefiting from immunotherapy (Figure 4E–H). We applied the risk score to stratify patients from an anti-PD-1/PD-L1 immunotherapy-treated NSCLC cohort (GSE135222, *n* = 27) into high- and low-risk groups. Kaplan–Meier analysis of progression-free survival (PFS) revealed a trend toward longer PFS in the low-risk group (Figure S1B); however, this difference did not reach statistical significance (*p* = 0.12). We further examined the expression differences of multiple canonical immune checkpoint genes between the low- and high-risk groups (Figure 4I,J). The results showed that the vast majority of inhibitory immune checkpoint genes and co-stimulatory molecule genes were expressed at significantly higher levels in the low-risk group than in the high-risk group. To further characterize the immune cell infiltration landscape across different risk groups, five distinct algorithms were employed to evaluate the tumor microenvironment (TME) composition of each sample in both groups (Figure 5A). Specifically, the CIBERSORT algorithm indicated that the low-risk group exhibited higher infiltration levels of CD8^+^ T cells, memory B cells, resting mast cells, and resting dendritic cells (Figure 5B). The ESTIMATE algorithm demonstrated that the stromal (*p* = 0.033), immune (*p* = 2 × 10^−7^), and ESTIMATE scores (*p* = 5.8 × 10^−5^) were markedly elevated in the low-risk group, reflecting higher immunogenicity, whereas the high-risk group exhibited a higher tumor purity score (*p* = 5.8 × 10^−5^), indicative of a prototypical “immune-cold tumor” phenotype (Figure 5C). Consistent with these findings, the MCP-counter algorithm revealed that T cells, CD8^+^ T cells, cytotoxic lymphocytes, NK cells, B lineage cells, and myeloid dendritic cells were all present at higher infiltration levels in the low-risk group (Figure 5D). Collectively, these results demonstrate that distinct risk groups exhibit markedly divergent immune landscapes, and patients in the high-risk group may harbor a more profoundly immunosuppressed immune infiltration status.

### 2.5. Multi-Omics Analysis of the Key Prognostic Gene RPS19BP1

**Figure 6:**
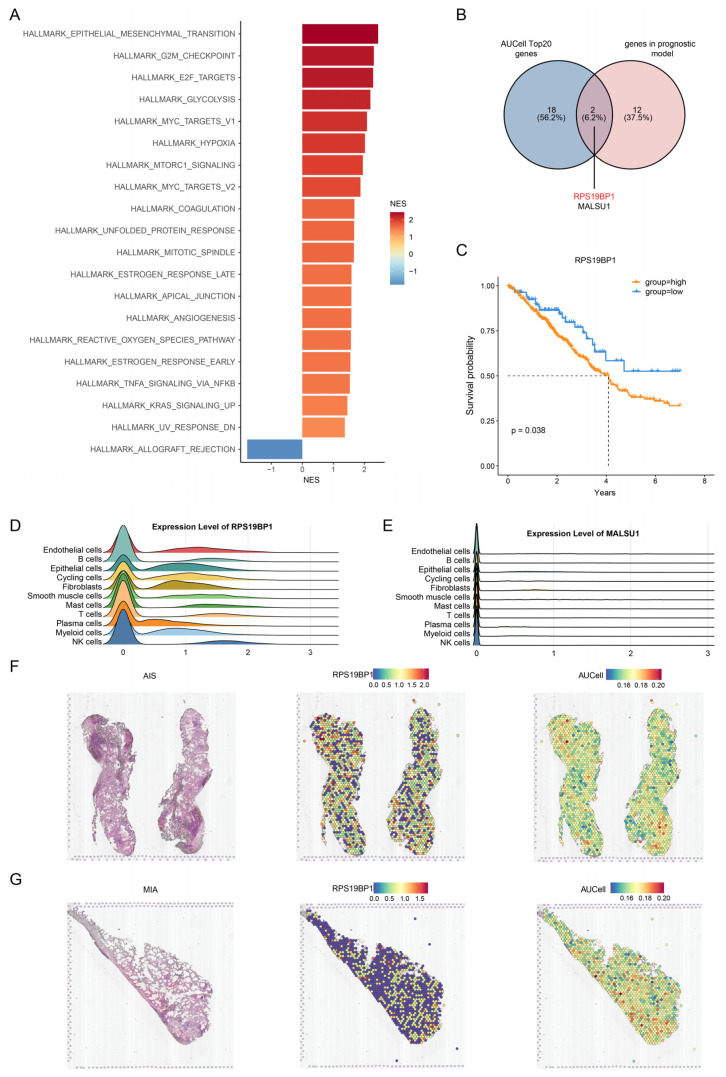
Clinical significance and multi-omics analysis of RPS19BP1. (**A**) The Gene Set Enrichment Analysis (GSEA) results of the high- and low-risk groups are presented by bar plots. (**B**) The Venn diagram shows the intersection of the top 20 AUCell correlation genes and the genes in the prognostic model. (**C**) Kaplan–Meier curves reveal the OS difference between *RPS19BP1* high- and low-expression groups in the TCGA dataset. (**D**,**E**) (D-*RPS19BP1*; E-*MALSU1*) The ridge plot shows the expression levels of two genes in different cell types. (**F**,**G**) (F-AIS; G-MIA) The heatmap demonstrated that AUCell and *RPS19BP1* exhibited similar spatial localization in tumor tissues at different progression stages.

**Figure 7:**
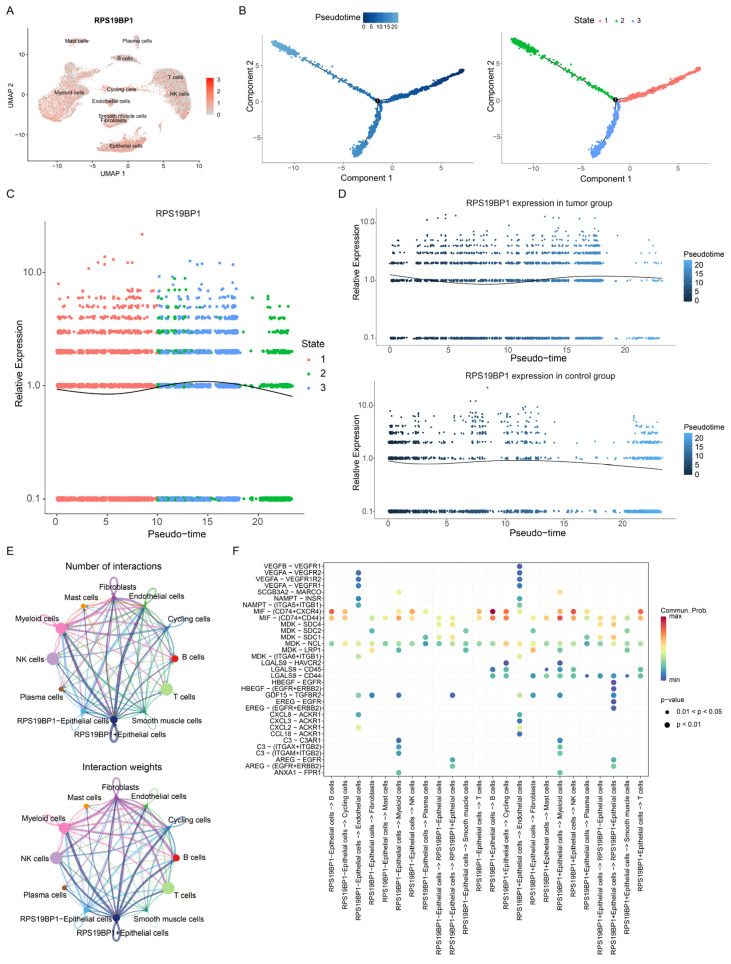
CellChat and Pseudotime Analysis of *RPS19BP1*. (**A**) UMAP plot of *RPS19BP1* expression in different cell types. (**B**) Pseudotime trajectory of epithelial cells reconstructed using Monocle2, colored by inferred developmental state and pseudotime. (**C**) Expression trajectory of *RPS19BP1* along pseudotime. (**D**) Expression trajectory of *RPS19BP1* along pseudotime in tumor tissue samples and normal tissue samples, respectively. (**E**) CellChat analysis of interaction count and strength, stratified by *RPS19BP1* expression. (**F**) The bubble plot shows the strength and probability of ligand-receptor pairs in cell interactions between *RPS19BP1*+ epithelial cells, *RPS19BP1*− epithelial cells and other cell types.

To investigate the differences in biological behavior between tumor tissues from distinct risk group, gene set enrichment analysis (GSEA) was performed on the bulk RNA-seq data of the TCGA-LUAD cohort stratified by risk group. The results revealed marked differences in the enrichment of multiple signaling pathways between the two groups (Figure 6A). In the high-risk group, cell proliferation-related pathways, including E2F target genes, the G2M checkpoint, mTORC1 signaling, and KRAS signaling, were significantly enriched, alongside invasion- and metastasis-related pathways such as epithelial–mesenchymal transition (EMT) and angiogenesis. In contrast, the allograft rejection pathway was enriched in the low-risk group, suggesting a more favorable immune infiltration profile. To further pinpoint key genes within the prognostic model, we intersected the top 20 genes most strongly correlated with the AUCell score with the genes constituting the prognostic model, yielding two core genes: RPS19BP1 and MALSU1 (Figure 6B). scRNA-seq analysis demonstrated that, in tumor samples, *RPS19BP1* exhibited higher expression levels than *MALSU1* across various cell types, particularly in epithelial cells (Figure 6D,E and Figure 7A). In the TCGA-LUAD cohort, patients with high RPS19BP1 expression had significantly poorer OS (*p* = 0.038) (Figure 6C). We therefore hypothesized that *RPS19BP1* may play an oncogenic role in LUAD, and subsequent analyses focused on lung cancer cells with elevated *RPS19BP1* expression. At the spatial transcriptomic level, we observed a degree of co-localization between the spatial distribution of *RPS19BP1* expression and the ribosome biogenesis AUCell score in both the adenocarcinoma in situ (AIS) and minimally invasive adenocarcinoma (MIA) stages of LUAD (Figure 6F,G), suggesting a potential role for *RPS19BP1* in early tumorigenesis and progression. Spot-level Spearman correlation analysis revealed a statistically significant positive correlation between *RPS19BP1* expression and ribosome biogenesis AUCell scores in both AIS (r = 0.118, *p* < 0.05) (Figure S1E) and MIA (r = 0.134, *p* < 0.05) (Figure S1F) sections. Pseudotime trajectory analysis of epithelial cells from the scRNA-seq data revealed that these cells diverged into three distinct cell states along the pseudotime trajectory (Figure 7B). Epithelial cells in the tumor group predominantly differentiated into state 3, whereas those in the control group largely adopted state 2. Furthermore, RPS19BP1 expression levels in the tumor group were consistently higher than those in the control group across all pseudotime points (Figure 7D) and were upregulated during the transition from state 1 to state 3 (Figure 7C), implying that *RPS19BP1* may have a potential association with tumor progression. CellChat analysis was performed to infer ligand–receptor communication probabilities between *RPS19BP1*+ and *RPS19BP1*− epithelial cells and other cell types within the TME. This computational analysis predicted that *RPS19BP1*+ epithelial cells exhibited a higher inferred interaction strength with other cell populations compared with their *RPS19BP1*− counterparts (Figure 7E). Furthermore, ligand–receptor pair analysis suggested that RPS19BP1+ epithelial cells displayed elevated communication probabilities for several outgoing signaling pathways, including MIF, VEGF, MDK, and LGALS9 (Figure 7F). Finally, we performed GSEA to compare pathway enrichment between *RPS19BP1*+ and *RPS19BP1*− epithelial cells. The results revealed that *RPS19BP1*+ epithelial cells were significantly enriched for pathways associated with ribosome, rRNA processing, and RNA translation, among others (Figure S1C,D). These computational findings are consistent with the possibility that *RPS19BP1* may exert its oncogenic effects through the activation of ribosome biogenesis and proliferation-related signaling cascades. Collectively, these findings suggest that *RPS19BP1* may participate in early tumor initiation and progression and may contribute to the establishment of an immunosuppressive and pro-invasive TME.

### 2.6. In Vitro Validation of the Oncogenic Role of RPS19BP1 in LUAD

**Figure 8:**
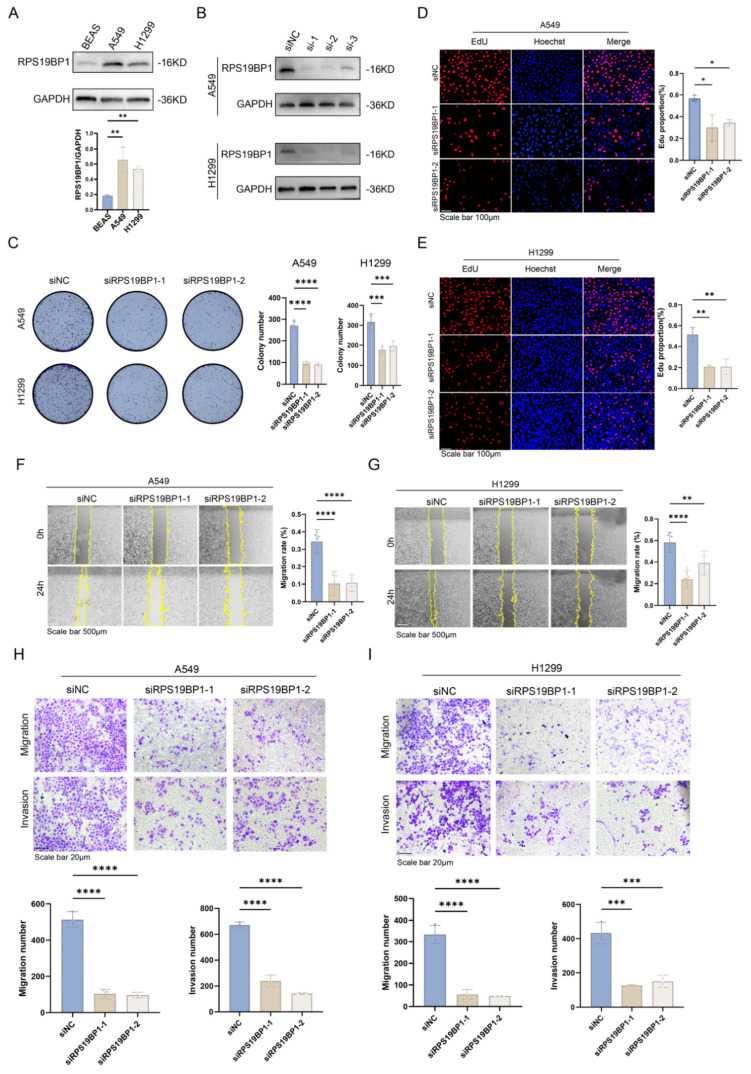
*RPS19BP1* is highly expressed in LUAD cell lines and promotes the proliferation of LUAD cell lines. (**A**) Western blot results indicated that the expression level of *RPS19BP1* in LUAD cell lines (A549, H1299) was higher than that in the human normal lung epithelial cell line (BEAS-2B) (*n* = 3). (**B**) Western blot verified the knockdown efficiency of siRNA (*n* = 3). (**C**) The colony formation assay revealed the effect of *RPS19BP1* knockdown on the proliferation of A549 and H1299 cells (*n* = 4). (**D**,**E**) (D-A549; E-H1299) The EdU assay confirmed the effect of *RPS19BP1* knockdown on LUAD cell proliferation (*n* = 3). (**F**,**G**) (F-A549; G-H1299) Microscopic images of the wound-healing assay showed the effect of *RPS19BP1* knockdown on the migration of LUAD cells (*n* = 6). (**H**,**I**) (H-A549; I-H1299) Microscopic images of the Transwell assay showed the effect of RPS19BP1 knockdown on the migration and invasion of LUAD cells (*n* = 4). Data are presented as mean ± SD from at least three independent biological replicates (*n* ≥ 3). The exact number of replicates for each experiment is indicated in the corresponding figure legend. (* means *p* < 0.05; ** means *p* < 0.01; *** means *p* < 0.001; **** means *p* < 0.0001).

To investigate the expression profile of *RPS19BP1* in LUAD, we assessed *RPS19BP1* protein expression levels by Western blotting in LUAD cell lines (including A549 and H1299) and the normal human bronchial epithelial cell line BEAS-2B. The results revealed that *RPS19BP1* was upregulated in LUAD cell lines relative to BEAS-2B cells (Figure 8A and Figure S3). To investigate the specific oncogenic function of *RPS19BP1* in LUAD, we silenced *RPS19BP1* expression in LUAD cell lines using specific siRNAs, and the knockdown efficiency was confirmed by Western blotting (Figure 8B and Figure S3). Colony formation assays (Figure 8C) demonstrated that *RPS19BP1* knockdown significantly reduced the clonogenic capacity of both A549 cells and H1299 cells (Figure 8C). EdU incorporation assays further revealed that the proportion of proliferating cells was markedly decreased following *RPS19BP1* silencing in A549 cells (Figure 8D) and H1299 cells (Figure 8E), suggesting that *RPS19BP1* may promote the proliferation of LUAD cells. Next, we examined the effect of *RPS19BP1* on the migration and invasion of LUAD cells. Wound healing assays demonstrated that *RPS19BP1* knockdown impeded wound closure in both A549 (Figure 8F) and H1299 cells (Figure 8G). Furthermore, Transwell assays revealed that silencing *RPS19BP1* significantly attenuated the migratory and invasive capacities of LUAD cells (Figure 8H,I).

## 3. Discussion

Lung cancer represents a global public health challenge that poses a grave threat to human health. In recent years, the emergence and advancement of scRNA-seq technology have enabled researchers to dissect both intratumoral and intertumoral heterogeneity of LUAD at the molecular level [18] and to delineate the reciprocal interactions between lung cancer cells and other cellular components within the TME. On the one hand, this has facilitated deeper exploration of the underlying biological mechanisms within tumor tissues; on the other hand, the elucidation of transcriptional signatures across distinct cell subpopulations has endowed RNA transcript-based prognostic biomarkers with greater biological interpretability and enhanced predictive power. Although a considerable number of studies have integrated scRNA-seq and bulk RNA-seq approaches to identify various biomarkers for LUAD—for instance, Song et al. constructed a B cell marker gene-based prognostic model for LUAD [19]—these prognostic models often lack external cohort validation of their predictive performance and wet-lab experimental verification of the biological functions of the identified genes, thus rendering their clinical relevance and reliability subject to further scrutiny.

Ribosome biogenesis-associated tumor heterogeneity represents a critical step in tumor initiation, progression, and metastasis [20]. Aberrations in this biological process manifest as alterations in rDNA copy number [21], changes in rRNA modifications [22], and heterogeneity of ribosomal proteins [23], ultimately giving rise to malignant “onco-ribosomes” [24]. Ribosome biogenesis also plays an important role in the progression of NSCLC. Yang et al. reported that downregulation of ribosomal protein L22 (*RPL22*) is associated with the development of NSCLC [25]. Another study by Yang et al. demonstrated that phosphorylation of ribosomal protein S3 (*RPS3*) confers radioresistance in NSCLC [26]. A study from Shandong First Medical University revealed that T cell differentiation protein 2 (*MAL2*) enhances cell proliferation by upregulating ribosome biogenesis activity in lung cancer cells [27]. Nevertheless, studies constructing LUAD prognostic models based on RBRGs remain notably scarce.

In this study, we first constructed a high-quality single-cell atlas using scRNA-seq data of LUAD and evaluated the ribosome biogenesis activity of individual cell subpopulations via the AUCell algorithm. The results revealed that epithelial cells, stromal cells, and various immune cell types in LUAD tissues exhibited higher AUCell scores compared with those in normal tissues, suggesting that ribosome biogenesis may contribute to both the maintenance of malignant phenotypes and the functional modulation of peritumoral immune cells. Subsequently, we identified RBRGs by selecting the top 100 genes most strongly correlated with the AUCell score and the upregulated DEGs in the high AUCell score group, and constructed a prognostic model based on bulk RNA-seq data from the TCGA-LUAD cohort. During the model development process, we observed that the prognostic model constructed using the full, untruncated survival data of all patients did not yet achieve the level of performance demonstrated by the model presented in this study. Considering the potential bias in survival data introduced by non-LUAD-related deaths during long-term follow-up, and given the sparse number of patients with overall survival exceeding 7 years, we elected to administratively censor the survival data at 7 years. In both the TCGA-LUAD cohort and the two external validation cohorts, the AUC values of the timeROC curves at 1, 2, and 3 years were greater than 0.7 in most cases, indicating that the model possesses robust predictive performance. Nevertheless, the 1-year AUC for GSE68571 was 67.93, reflecting a relatively modest performance. This may be attributable to inter-cohort heterogeneity, as well as potential batch effects arising from differences in sequencing platforms, which could compromise the model’s predictive accuracy. Additionally, the limited sample size of the GSE68571 cohort may have contributed to instability in the AUC estimate.

The prognostic model established in this study comprises 14 core genes, namely *GPRC5A*, *LGALS3*, *CD9*, *TM4SF1*, *GDF15*, *WFDC2*, *HLA-DRB5*, *VAMP8*, *TIMP1*, *CSTB*, *CD68*, *XRCC5*, *RPS19BP1*, and *MALSU1*. *GPRC5A* encodes an orphan G protein-coupled receptor. Although *GPRC5A* was initially identified as a lung-specific tumor suppressor gene whose loss promotes lung tumorigenesis, emerging evidence suggests that it possesses context-dependent dual functions in NSCLC [28]. Specifically, Jin et al. demonstrated that under a wild-type p53 background, *GPRC5A* suppresses proliferation, whereas in the presence of p53 mutation *GPRC5A* is upregulated and acquires pro-proliferative functions, with its high expression being associated with worse OS [29]. Furthermore, *GPRC5A* has been demonstrated to promote tumor metastasis through diverse signaling pathways in other cancer types, including breast cancer, gallbladder cancer, and esophageal cancer [30,31,32]. Taken together, these findings support the notion that *GPRC5A* plays a complex, context-dependent role in LUAD that warrants further investigation. *LGALS3* is implicated in immune regulation and cell adhesion, and in lung cancer, it has been shown to facilitate tumor progression by driving the generation of immunosuppressive macrophages [33]. *TM4SF1* is associated with angiogenesis and tumor invasion; in NSCLC, *TM4SF1* activates the AKT pathway to promote lung cancer cell proliferation and confer chemoresistance [34]. *TIMP1* promotes cell cycle progression and is recognized as a biomarker of poor prognosis in lung cancer [35]. *XRCC5*, also known as *Ku80*, participates in the DNA damage repair process and has been widely demonstrated to be closely linked to radioresistance and chemoresistance in NSCLC [36,37]. *MALSU1* regulates mitochondrial ribosome biogenesis and translational activity and has been implicated in chemoresistance in breast cancer [38]; however, its role in lung cancer has yet to be elucidated. *CD9* is a tetraspanin family member that regulates cell adhesion, migration, and membrane fusion. Its high expression has been shown to be associated with brain metastasis in NSCLC [39], although the underlying mechanism remains largely unknown. *GDF15* has been reported to alleviate anemia by mitigating ribosomal stress [40], yet its functional role in lung cancer has not been elucidated. *CD68* is a classical macrophage marker involved in biological processes such as antigen processing. *WFDC2* has previously been proposed as a prognostic biomarker for lung cancer [41]. Mutations in *HLA-DRB5* have been linked to EGFR-TKI resistance in NSCLC patients [42]. *VAMP8* has been implicated in NSCLC progression through its regulation of autolysosome maturation [43]. However, whether these genes are functionally connected to ribosome biogenesis has not been investigated. Future studies should further explore these genes, as they may represent novel components within the ribosome biogenesis regulatory network.

Following validation, the prognostic model demonstrated robust performance in predicting the 1-, 2-, and 3-year prognosis of LUAD patients. Upon stratifying patients by risk score, we observed a relatively weak correlation between TMB and the risk score across the high- and low-risk groups; nevertheless, computational analyses suggested that the high-risk group may exhibit a poorer response to immunotherapy, suggesting that the TME of the high-risk group may represent an immunosuppressive “cold tumor” phenotype. However, the correlation between risk score and TMB was modest (r = 0.104), suggesting that TMB represents only one of many factors contributing to the prognostic value of the risk score. The immunotherapy response prediction analysis was constrained by the small sample size of the available anti-PD-1/PD-L1-treated cohort (GSE135222, *n* = 27), which limited the statistical power to detect significant differences in PFS between risk groups. While a trend toward longer PFS was observed in the low-risk group (*p* = 0.12), this finding should be regarded as hypothesis-generating rather than confirmatory. Validation in larger, independent cohorts of NSCLC patients treated with immune checkpoint blockade is warranted to substantiate the predictive capacity of the risk score for immunotherapy response. Comparison of immune checkpoint molecule expression between the two risk groups revealed that the high-risk group displayed lower expression levels of inhibitory immune checkpoint molecules, which may be associated with a potential reduced response rate to ICB therapy. Further TME characterization using multiple algorithms demonstrated that the high-risk group exhibited diminished immune cell infiltration, with CD8+ T cell infiltration being notably lower than that in the low-risk group. Conversely, the TME of the low-risk group appeared to be more permissive to effective immune responses and immune surveillance, accompanied by higher expression levels of various ICB targets. Moreover, multiple pro-tumorigenic pathways were enriched in the high-risk group, and the malignant biological processes underlying these pathways may account for the poor prognosis observed in high-risk patients.

At the molecular level, we selected *RPS19BP1*, a core gene associated with ribosome biogenesis, for subsequent multi-omics analysis and in vitro experiments to validate its role in the proliferation and invasion of LUAD cells. *RPS19BP1*, also known as *AORS*, was first reported by Kim et al. in 2007 [44]. They discovered that in human colorectal adenocarcinoma cell lines, RPS19BP1 enhances Silent Information Regulator 1 (*SIRT1*)-mediated deacetylation of p53 through direct binding to *SIRT1*, thereby suppressing tumor cell apoptosis. In 2013, Knight et al. demonstrated that *RPS19BP1* is essential for the biogenesis of the 40S ribosomal subunit, establishing it as a critical factor in ribosome biogenesis [45]. However, the impact of RPS19BP1 on malignant phenotypes of LUAD cells, including proliferation and invasion, has remained uncharacterized. In our scRNA-seq data, we observed that epithelial cells in LUAD tissues exhibited higher *RPS19BP1* expression levels than those in normal tissues across all stages of cell differentiation, suggesting a potential association with LUAD progression. CellChat analysis predicted enhanced ligand-receptor interactions involving RPS19BP1+ cells, suggesting their potential role in shaping an immunosuppressive TME. GSEA inferred that *RPS19BP1* may exert its oncogenic effects through the activation of ribosome biogenesis and proliferation-related signaling cascades. Spatial transcriptomic analysis corroborated the co-localization of *RPS19BP1* expression and ribosome biogenesis activity, particularly during the early stages of LUAD progression. It should be noted that the spot-level correlation between *RPS19BP1* expression and AUCell scores, although statistically significant, was relatively weak (r ≈ 0.10). This is likely attributable to the multicellular composition of individual spatial transcriptomic spots, which averages expression signals across heterogeneous cell populations and attenuates gene-level correlations. Using in vitro experiments, we further provided preliminary evidence validating the oncogenic role of *RPS19BP1* in LUAD. Intriguingly, although *RPS19BP1* has been previously reported to exert its oncogenic effects through the suppression of p53 [44], knockdown of *RPS19BP1* in the p53-null H1299 cell line still suppressed its proliferative, migratory, and invasive capacities. This observation suggests that *RPS19BP1* may sustain the malignant phenotype of LUAD cells through mechanisms that are at least partly independent of p53. However, the present study did not directly demonstrate whether *RPS19BP1* silencing alters rRNA synthesis, ribosomal protein composition, or global protein translation rates; therefore, the mechanistic link between *RPS19BP1* and ribosome biogenesis requires further investigation.

Although numerous studies have claimed to construct LUAD prognostic signatures using scRNA-seq and bulk RNA-seq, the majority of these studies rely primarily on bulk RNA-seq, with all prognostic model construction steps performed exclusively on bulk RNA-seq data from TCGA/GEO databases, while scRNA-seq data are used only for basic gene expression profiling. The substantive distinction between our study and these LUAD prognostic models lies in the application of the AUCell algorithm to first establish, at the single-cell level, that ribosome biogenesis is enhanced in tumor samples. The RBRGs were subsequently identified based on scRNA-seq analysis, ensuring a more direct and robust connection to ribosome biogenesis. Furthermore, our model achieved AUC values greater than 0.7 at the majority of survival time points across two independent external validation cohorts. By contrast, many published LUAD prognostic signatures either lack external validation entirely—such as the B cell marker-based LUAD prognostic model constructed by Song et al. [19]—or were validated in only a single external cohort, such as the lactylation-related gene-based LUAD prognostic model developed by Gao et al. [46]. Among studies that performed validation in multiple independent cohorts, the reported AUC values of the prognostic models mostly range between 0.6 and 0.7, as exemplified by the study published by Zhang et al. [47]. Thus, the predictive performance of our model compares favorably with the existing literature. Finally, beyond constructing a prognostic model, our study further explored the key gene RPS19BP1 through pseudotime trajectory analysis, CellChat analysis, and functional in vitro experiments, thereby improving biological and clinical value.

Although this study constructed a robust prognostic model with favorable predictive performance through the integration of multi-omics RNA-seq data and demonstrated consistent efficacy across multiple external validation cohorts, several limitations warrant consideration. First, this study is a retrospective investigation based on publicly available databases, and the findings are therefore inherently dependent on the quality and reliability of these repositories. The external validation cohorts (GSE68571, *n* = 86; GSE8894, *n* = 63) were relatively small, which may affect the precision of AUC and C-index estimates. The validation datasets were generated on microarray platforms, whereas the training cohort (TCGA-LUAD) used RNA-seq, and cross-platform differences in gene expression quantification may contribute to the modest AUC values observed in the external cohorts. The robustness and clinical utility of the model in real-world settings remain to be validated through multicenter prospective trials and relatively larger cohorts in the future. Second, although the prognostic model associates the high-risk group with an immunosuppressive TME, the specific mechanisms by which the model-derived risk stratification influences the TME were not elucidated in this study. In conclusion, future efforts should focus on validating our prognostic model in more diverse and heterogeneous cohorts to ensure its sufficient clinical relevance. Furthermore, additional in vitro and in vivo studies that include assays assessing rRNA synthesis, ribosomal protein composition, and global protein translation rates after *RPS19BP1* silencing are warranted to comprehensively delineate the precise downstream signaling pathways and the underlying molecular mechanisms through which *RPS19BP1* exerts its effects in LUAD cells. The functional validation of *RPS19BP1* relied on transient siRNA-mediated knockdown; although two independent siRNA sequences were used to minimize potential off-target effects, stable knockdown approaches, rescue experiments, and xenograft or metastasis models would provide more definitive confirmation of target specificity and represent important directions for future investigations.

## 4. Materials and Methods

### 4.1. Data Acquisition and Initial Processing

In this study, single-cell RNA sequencing (scRNA-seq) data for LUAD were obtained from the GSE131907 database (https://www.ncbi.nlm.nih.gov/geo/, accessed on 15 August 2025) [48], comprising 11 primary tumor tissues and 11 matched distant normal lung tissues. The training cohort included RNA expression patterns of 495 patients with LUAD and corresponding clinical information from The Cancer Genome Atlas (TCGA) database (https://portal.gdc.cancer.gov/, accessed on 15 August 2025). Validation sets were derived from GSE68571 (86 patients) [49], GSE8894 (63 patients) [50] and GSE135222 (27 patients) [51] expression profiles from the Gene Expression Omnibus (GEO) database (https://www.ncbi.nlm.nih.gov/geo/, accessed on 15 August 2025). In subsequent analyses, samples with missing clinical information or an overall survival (OS) time of 0 were excluded. Given that the time-dependent ROC analyses in this study were designed to evaluate 1-, 2-, and 3-year prognostic performance, and a recent study used 7-year survival as the final time point in time-dependent ROC analyses [52], a maximum follow-up duration of 7 years was set, and all study subjects were administratively censored at the 7-year follow-up time point. Only a small number of patients were excluded due to the 7-year censoring cutoff (TCGA: 27/495; GSE8894: 5/63; GSE68571: 10/86), thereby ensuring that the truncated cohorts remained representative of the original study populations. To ensure data comparability across datasets, all bulk RNA-seq gene expression values were normalized to transcripts per million (TPM), calculated from raw counts corrected for gene length and sequencing depth, except for differential expression analysis, for which raw count data were used. Before statistical analysis, all bulk RNA-seq expression data were subjected to log2 transformation to normalize the data distribution and improve the validity of downstream statistical tests. This study incorporated one sample of LUAD spatial Transcriptomics GSE189487 (https://www.ncbi.nlm.nih.gov/geo/, accessed on 2 January 2026) [53]. A total of 325 genes were selected based on the GOBP_RIBOSOME_BIOGENESIS.v2023.2.Hs gene set, which was obtained from the Molecular Signatures Database (MSigDB) (https://www.gsea-msigdb.org/gsea/msigdb/index.jsp, accessed on 17 August 2025).

### 4.2. scRNA-Seq Data Processing

The scRNA-seq data were analyzed using the “Seurat” R package. Quality control was performed by retaining cells expressing 200–5000 genes, containing 200–30,000 unique molecular identifier (UMI) counts, with ≤30% mitochondrial genes and ≤5% hemoglobin genes. Data normalization was performed using the LogNormalize function implemented in Seurat with the following parameters: scale.factor = 10,000 and normalization.method = “LogNormalize”. We selected the top 2000 variable genes using the vst algorithm and performed scaling and principal component analysis (PCA). Batch effects were corrected using Harmony. “FindNeighbors” and “FindClusters” functions were applied, resulting in a resolution of 1.2. Cell clusters were manually annotated based on the expression of canonical cell-type-specific marker genes. These marker genes were identified using “FindAllMarkers” in Seurat, and the results were visualized using “visCluster” in “ClusterGVis” R package (Zhang, 2022; https://github.com/junjunlab/ClusterGVis, accessed on 5 February 2026). Ribosome biogenesis activity scores for individual cells were computed using the “AUCell” R package [54]. Subsequently, cells were stratified into high- and low-ribosome biogenesis groups according to the median AUCell score. In order to capture as many genes related to ribosome biogenesis as possible, differential analyses were conducted to identify differentially expressed genes (DEGs) (adjust *p* value < 0.05, log2 fold change(FC) > 0) between high- and low-ribosome biogenesis groups, resulting in the selection of 165 upregulated DEGs for further exploration. Additionally, a correlation analysis was conducted to identify genes that are strongly associated with ribosome biogenesis activity. Following the method described by Huang et al. [55], the top 100 genes with the strongest correlation to the AUCell score were selected for further investigation. Considering that ribosome biogenesis is typically activated rather than suppressed and upregulated genes are functionally more likely to be directly involved in the aberrant regulation of ribosome biogenesis in tumors, the upregulated DEGs (*n* = 165) and top 100 genes (*n* = 100) represent those genes that have the most significant impact on ribosome biogenesis activity which we called RBRGs (*n* = 262).

### 4.3. Functional Enrichment Analysis

Gene Ontology (GO) biological process (BP) enrichment analysis was performed to verify the potential functions of each cell cluster that we manually annotated. To investigate the biological pathways specifically enriched in the high-ribosome biogenesis group, we also performed GO and Kyoto Encyclopedia of Genes and Genomes (KEGG) enrichment analyses using the RBRGs. In addition, Gene Set Enrichment Analysis (GSEA) [56] was conducted to identify significantly enriched pathways using the differential gene expression profiles from bulk RNA-seq (high-risk vs. low-risk groups) and scRNA-seq (*RPS19BP1*+ epithelial cells vs. *RPS19BP1*− epithelial cells).

### 4.4. Construction and Validation of the Prognostic Model

To avoid zero expression values for certain genes across samples, we normalized the raw data using log2(TPM + 1) transformation. Univariate Cox proportional hazards regression analysis was performed on 262 RBRGs identified from scRNA-seq data to screen for genes significantly associated with patient overall survival (*p* < 0.1). Subsequently, a combination of least absolute shrinkage and selection operator (LASSO) regression and multivariate Cox proportional hazards regression analysis was employed to further refine gene selection and determine risk coefficients closely correlated with prognosis. LASSO regression with 10-fold cross-validation was performed using the cv.glmnet() function to select the optimal penalty parameter λ and identify the most informative prognostic genes. The value of λ that minimized the partial likelihood deviance (lambda.min) was selected as the optimal penalty parameter. Cross-validation was performed with 10 folds and a maximum of 100,000 iterations, and the random seed 4 was fixed to ensure reproducibility. Genes with non-zero coefficients in the LASSO model were regarded as potential prognostic biomarkers. Finally, the risk score for each LUAD patient was calculated using the coefficients derived from the multivariate Cox regression analysis. In the TCGA-LUAD training cohort, 172 deaths were recorded during follow-up. With 14 genes included in the final multivariable Cox model, the events-per-variable ratio (EPV) was 12.3 (172/14), exceeding the commonly recommended threshold of 10, suggesting that the risk of overfitting was within an acceptable range. In the TCGA-LUAD cohort, patients were divided into high-risk and low-risk groups according to the median value of the calculated risk score derived from the prognostic signature. Kaplan–Meier survival curves were constructed for the two risk groups, and the log-rank test was applied to evaluate the statistical significance of survival differences. The prognostic performance of the signature was assessed using time-dependent receiver operating characteristic (timeROC) curves. Furthermore, the robustness and predictive efficacy of the prognostic model were independently validated in two external GEO datasets, with survival analysis and timeROC-derived area under the curve (AUC) calculation performed for verification. In addition to time-dependent timeROC analysis, the discriminative performance of the prognostic model was quantified using Harrell’s concordance index (C-index). These metrics were independently validated in two external GEO datasets, where survival analysis, timeROC-derived AUC, and C-index calculations were performed to confirm the robustness and generalizability of the signature.

### 4.5. Comparison of Clinical Factors with the Risk Score and Construction of a Nomogram

Patients with missing clinical data in the TCGA-LUAD cohort were excluded, resulting in a final set of 338 patients with complete clinical annotations. Univariate Cox regression analysis was performed to evaluate the association of age, sex, T stage, N stage, M stage, and the risk score with overall survival. Among these variables, T stage, N stage, and the risk score exhibited *p* < 0.05 and were subsequently entered into a multivariate Cox regression analysis, in which all three variables remained statistically significant (*p* < 0.05). A nomogram integrating T stage, N stage, and the risk score was constructed using the “rms” R package to predict 1-, 2-, and 3-year overall survival probabilities. The calibration of the nomogram was assessed by randomly dividing patients into three groups, repeating this procedure 1000 times, and plotting the mean predicted versus observed survival probabilities for each time point. Decision curve analysis (DCA) was performed to evaluate the net clinical benefit of the nomogram and of different combinations of T stage, N stage, and the riskscore at 1, 2, and 3 years.

### 4.6. Tumor Mutation Burden Analysis

The “maftools” R package [57] was utilized to calculate the tumor mutational burden (TMB) between high-risk and low-risk groups in the TCGA-LUAD cohort. The landscape of the TMB in different groups was plotted using a waterfall chart. The correlation between the risk score and tumor mutation burden (TMB) was evaluated using Pearson correlation analysis. TMB values were log2-transformed (log2(TMB + 1)) to improve data distribution normality.

### 4.7. Immune Cell Infiltration and Potential Immunotherapy Response Prediction

The infiltration status of immune cells in the TME was estimated using six algorithms, including CIBERSORT, MCP-counter, EPIC, ESTIMATE, quanTIseq, and immunophenoscore (IPS), via the “IOBR” R package [58]. The results were visualized using the “pheatmap” and “ggplot2” R packages. Additionally, we compared the expression levels of key immune checkpoint genes between the high- and low-risk groups. The immunosuppressive checkpoints selected for analysis included *LAG3*, *KIR3DL1*, *TIGIT*, *CD160*, *CD274*, *BTLA*, *CTLA4*, *LAIR1*, *HAVCR2*, *ADORA2A*, *CD200R1*, *BTNL2*, *PDCD1*, *CD276*, *IDO2*, *LGALS9*, and *PDCD1LG2*. The immune-activated checkpoints selected for analysis included *CD86*, *CD80*, *TNFSF18*, *TNFSF14*, *TNFSF9*, *CD40LG*, *ICOS*, *CD48*, *TNFRSF8*, *CD28*, *TNFRSF25*, *CD27*, *CD40*, and *TNFRSF14*. To further evaluate the potential association between risk stratification and immunotherapy response, the Tumor Immune Dysfunction and Exclusion (TIDE) algorithm [59] was applied to assess treatment response patterns between the two risk groups. We utilized an NSCLC cohort of patients treated with anti-PD-1/PD-L1 immunotherapy (GSE135222) and stratified the patients into high- and low-risk groups based on the risk score. Kaplan–Meier survival analysis was then performed to evaluate whether progression-free survival (PFS) differed between the two risk groups. Between-group differences in immune cell infiltration, ESTIMATE scores, MCP-counter, IPS, expression levels of key immune checkpoint genes and TIDE scores were assessed using the Wilcoxon rank-sum test. Multiple-testing correction was performed using the Benjamini–Hochberg method, with an adjusted *p* value < 0.05 considered statistically significant. All immune infiltration analyses were conducted using the default parameter settings of the respective algorithms via the IOBR R package.

### 4.8. Identification of DEGs Between the High-Risk and Low-Risk Groups

Differential expression analysis between high-risk and low-risk groups was performed. Raw count data from the TCGA-LUAD cohort were analyzed using the “DESeq2” R package. A DESeqDataSet object was constructed from the raw count matrix and the two-group condition variable (high-risk vs. low-risk). Normalization and differential expression testing were performed using the default DESeq2 pipeline with the Wald test. Genes with |log2 FC| > 1 and Benjamini–Hochberg adjusted *p* value < 0.05 were considered significantly differentially expressed.

### 4.9. Spatial Transcriptomics Data Analysis

Spatial transcriptomics data were obtained from the publicly available dataset GSE189487. The data were generated using the 10× Genomics Visium spatial gene expression platform, with sequencing performed on the Illumina HiSeq 3000 system (Illumina Inc., GPL21290, San Diego, USA). Each tissue section was captured on a Visium slide with capture spots of 55 µm in diameter and a center-to-center distance of 100 µm between adjacent spots. The adenocarcinoma in situ (AIS) section contained 1700 spots, and the minimally invasive adenocarcinoma (MIA) section comprised 1140 spots. Spatial transcriptomic data were analyzed using the “Seurat” R package. Subsequent data normalization and variance stabilization were conducted using the SCTransform algorithm. PCA was then performed for dimensionality reduction. Based on the top 15 principal components, we constructed a nearest neighbor graph and performed unsupervised spot clustering using “FindNeighbors” and “FindClusters”. The “AUCell” R package [54] was utilized to calculate ribosome biogenesis activity scores for each spot in the spatial transcriptomic dataset. The previously defined ribosome biogenesis gene set was used as the input gene signature. AUCell calculates an area under the curve (AUC) score for each spot based on the ranking of gene expression values, thereby quantifying the enrichment of genes related to ribosome biogenesis relative to the global transcriptome within each spatial spot. The resulting AUCell scores were then projected onto the spatial coordinates of the tissue sections to visualize the distribution of ribosome biogenesis activity. The resulting AUC values were visualized using spatial feature plots to illustrate the spatial distribution of ribosome biogenesis activity. The spatial expression of the targeted gene *RPS19BP1* was also visualized for reference. Spot-level Spearman correlation analysis was performed between *RPS19BP1* expression and ribosome biogenesis AUCell scores to quantitatively evaluate their spatial association.

### 4.10. Single-Cell Pseudotime and Cell-to-Cell Interaction Analysis

scRNA-seq data of lung cancer cells were analyzed using the “Monocle2” R package [60] to explore the association between *RPS19BP1* expression and cellular pseudotime trajectories. The “CellChat” R package [61] was employed to identify and quantify intercellular communication among distinct cell types in the single-cell dataset.

### 4.11. Western Blot

RIPA lysis buffer (Beyotime, P0013B, Shanghai, China) was utilized to lyse the cells. Protein concentrations were quantified using the BCA method. Samples were subjected to electrophoresis on an 8% sodium dodecyl sulfate polyacrylamide gel and subsequently transferred onto PVDF membranes. The membranes were blocked with 5% (*w*/*v*) non-fat dried milk in Tris-buffered saline containing 0.1% Tween-20 (TBST) for 1 h at room temperature to block non-specific binding sites. The membranes were then incubated overnight at 4 °C with primary antibodies against RPS19BP1 and GAPDH all sourced from China ABclonal Corporation (RPS19BP1: A13231; GAPDH: AC002, Wuhan, China). Subsequently, the membranes were incubated with goat anti-rabbit IgG or anti-mouse IgG for 1 h at room temperature. The immunoreactivity was visualized using the SuperKine™ West Femto Maximum Sensitivity Substrate (Abbkine, Wuhan, China), with GAPDH serving as a loading control. Western blot analysis was employed to assess the protein levels of RPS19BP1. Relative protein expression levels were quantified based on the intensity of the corresponding bands.

### 4.12. Cell Line Culture

We acquired the BEAS-2B human lung epithelial cell line and the A549 and H1299 human LUAD cell line from the Chinese Academy of Science Committee on Type Culture Collection Cell Bank (Shanghai, China). It was necessary to culture A549 cells in DMEM/F12 medium (Gibco; Thermo Fisher Scientific, Inc., Waltham, MA, USA) and to culture BEAS-2B cells and H1299 cells in 1640 medium (Gibco; Thermo Fisher Scientific, Inc., Waltham, MA, USA), both of which contained 10% fetal bovine serum (Procell, 164210, Wuhan, China) and 1% penicillin-streptomycin (Beyotime, C0224, Shanghai, China). The culture conditions were maintained to ensure optimal cell growth and viability. This involved setting the incubator temperature at 37 °C, maintaining a CO2 concentration of 5% and ensuring a humidity level of 95%.

### 4.13. Cell Transfection of Small Interfering RNA

Small interfering RNA (siRNA) transfection was facilitated by Lipo2000 reagent from Invitrogen, Waltham, MA, USA, conducted in accordance with the manufacturers’ protocols. Coverslips positioned in six-well plates were inoculated with LUAD cells, and transfection with siRNA was carried out 48 h post-seeding. The specific target sequences utilized are detailed as follows: si-RPS19BP1-1# Sequence (5′ -> 3′).

Sense CCGGAAAGGAACGUAUCUGUUtt

Antisense AACAGAUACGUUCCUUUCCGGtt

si-RPS19BP1-2# Sequence (5′ -> 3′).

Sense GACCACCUCAGAGUAAACCUGtt

Antisense CAGGUUUACUCUGAGGUGGUCtt

si-RPS19BP1-3# Sequence (5′ -> 3′).

Sense UGAGCCAGCAGAUUUUGCGCCtt

Antisense GGCGCAAAAUCUGCUGGCUCAtt

### 4.14. EdU Incorporation Assay

EdU assay was undertaken with BeyoClick™ EdU Cell Proliferation Kit with Alexa Fluor 594 (Beyotime, C0078S, Shanghai, China). After washing in PBS, EdU solution was used to incubate cells for 2 hours. Cell nuclei were then stained with Hoechst solution. After washing, samples were observed with an inverted microscope (Olympus, Tokyo, Japan).

### 4.15. Wound-Healing Assay

When LUAD cells reached 90% confluence, they were seeded into six-well culture dishes. A linear scratch wound was created across the cell monolayer using the tip of a 200 μL pipette. Subsequently, A549 cells were cultured in fetal bovine serum (FBS) free medium, while H1299 cells were maintained in medium supplemented with 2% FBS. The cell monolayer was gently rinsed with phosphate-buffered saline (PBS) to remove cell debris and floating cells. Images of each scratch wound were captured using an inverted microscope immediately after wounding (0 h) and again at 24 h post-wounding to assess cell migration ability.

### 4.16. Transwell Assay

Transwell chambers (Nest, 8 μm pore size) with 24 wells were used to evaluate the invasion and migration abilities of LUAD cells. For the matrix gel invasion assay, Transwell membranes (MCE, HY-K6002, Monmouth Junction, NJ, USA) were pre-coated with matrix gel prior to use. A total of 1 × 10^4^ cells were seeded into the upper chamber of each Transwell insert, which was filled with 200 μL of serum-free medium to maintain cell viability and growth. Meanwhile, the lower chamber was supplemented with 600 μL of medium containing 10% FBS as a chemoattractant. After 24 h of incubation, cells that had not migrated or invaded (remaining on the upper surface of the membrane) were gently removed. Cells that had migrated or invaded through the membrane to the lower surface were fixed with 4% solution of paraformaldehyde and stained with 0.1% crystal violet. Images of the stained cells were captured using an inverted microscope (Olympus, Tokyo, Japan) for subsequent quantitative analysis.

### 4.17. Colony Formation Assay

In the colony formation assay, LUAD cells were plated at a density of 1 × 10^3^ cells per well within six-well plates. These plates were then incubated at a temperature of 37 °C with an atmosphere containing 5% carbon dioxide, for a period of 14 days. Once the incubation period was completed, the colonies were fixed using a 4% solution of paraformaldehyde. Subsequently, they were stained with a 0.1% solution of crystal violet. Colonies containing more than 50 cells were meticulously counted in each well to ensure accuracy. The experiments were conducted in triplicate.

### 4.18. Statistical Analysis

Initial data processing, along with statistical analysis and result visualization, was performed using R software (version 4.2.2) and GraphPad Prism 10 (version 10.6). Data are presented as mean ± standard deviation from at least three independent experiments. Spearman correlation analysis was employed to assess the relationship between two continuous variables. For categorical variables, comparisons were conducted using the Chi-square test. For continuous variables, either the Wilcoxon rank-sum test or *t*-test was used for group comparisons. Multiple group comparisons were analyzed using one-way ANOVA with Tukey’s post hoc test. All statistical tests were two-tailed, and statistical significance was defined as a *p*-value < 0.05 unless otherwise specified. Specific statistical methods for each experiment are detailed in the corresponding figure legends.

## 5. Conclusions

In this study, we constructed a ribosome biogenesis-based prognostic model for LUAD using scRNA-seq data. This model effectively stratifies patients by risk, demonstrates robust predictive performance, and is associated with distinct immune infiltration patterns, suggesting its potential utility in guiding immunotherapy decisions. These findings may aid in identifying patients with unfavorable prognosis, provide potential therapeutic targets centered on the biological process of ribosome biogenesis, and broaden our understanding of ribosome biogenesis in LUAD.

## Data Availability

The original contributions presented in this study are included in the article. Further inquiries can be directed to the corresponding authors. The analysis code is available from the corresponding authors upon reasonable request.

## Supplementary Materials

The following supporting information can be downloaded at https://www.mdpi.com/article/10.3390/ijms27135864/s1.

## Abbreviations

AIS	adenocarcinoma in situ
ANOVA	analysis of variance
AORS	active regulator of SIRT1
AUC	area under the curve
BCA	bicinchoninic acid
BP	biological process
CIBERSORT	cell-type identification by estimating relative subsets of RNA transcripts
CSTB	cystatin B
DEG	differentially expressed gene
DMEM	Dulbecco’s Modified Eagle Medium
E2F	E2 transcription factor
EdU	5-ethynyl-2′-deoxyuridine
EMT	epithelial–mesenchymal transition
EPIC	estimating the proportions of immune and cancer cells
ESTIMATE	estimation of stromal and immune cells in malignant tumor tissues using expression data
FBS	fetal bovine serum
FC	fold change
G2M	G2/mitosis
GAPDH	glyceraldehyde-3-phosphate dehydrogenase
GDF15	growth differentiation factor 15
GEO	Gene Expression Omnibus
GO	Gene Ontology
GPRC5A	G protein-coupled receptor family C group 5 member A
GSEA	gene set enrichment analysis
HLA-DRB5	human leukocyte antigen DRB5
ICB	immune checkpoint blockade
IgG	immunoglobulin G
IOBR	immuno-oncology biological research
IPS	immunophenoscore
KEGG	Kyoto Encyclopedia of Genes and Genomes
LASSO	least absolute shrinkage and selection operator
LGALS3	galectin-3
LGALS9	galectin-9
LUAD	lung adenocarcinoma
LUSC	lung squamous cell carcinoma
MAL2	T cell differentiation protein 2
MALSU1	mitochondrial assembly of ribosomal large subunit 1
MCP-counter	microenvironment cell populations-counter
MDK	midkine
MIA	minimally invasive adenocarcinoma
MIF	macrophage migration inhibitory factor
MSigDB	Molecular Signatures Database
mTOR	mammalian target of rapamycin
mTORC1	mammalian target of rapamycin complex 1
NF-κB	nuclear factor kappa-light-chain-enhancer of activated B cells
NK	natural killer
NSCLC	non-small cell lung cancer
OS	overall survival
PBS	phosphate-buffered saline
PCA	principal component analysis
PVDF	polyvinylidene difluoride
quanTIseq	quantification of the tumor immune contexture from RNA-seq data
RBIS	ribosome biogenesis factors
RBRG	ribosome biogenesis-related gene
rDNA	ribosomal DNA
RIPA	radioimmunoprecipitation assay
RPL22	ribosomal protein L22
RPS3	ribosomal protein S3
RPS19BP1	ribosomal protein S19 binding protein 1
rRNA	ribosomal RNA
SCENIC	single-cell regulatory network inference and clustering
scRNA-seq	single-cell RNA sequencing
SCTransform	single-cell transform
SIRT1	silent information regulator 1
siRNA	small interfering RNA
SOD1	superoxide dismutase 1
STAT3	signal transducer and activator of transcription 3
TBST	Tris-buffered saline containing 0.1% Tween-20
TCGA	The Cancer Genome Atlas
TIDE	tumor immune dysfunction and exclusion
timeROC	time-dependent receiver operating characteristic
TIMP1	tissue inhibitor of metalloproteinases 1
TM4SF1	transmembrane 4 L six family member 1
TMB	tumor mutational burden
TME	tumor microenvironment
TP53	tumor protein 53
TPM	transcripts per million
TXN	thioredoxin
UMAP	uniform manifold approximation and projection
UMI	unique molecular identifier
VAMP8	vesicle-associated membrane protein 8
VEGF	vascular endothelial growth factor
WDR18	WD repeat domain 18
WFDC2	WAP four-disulfide core domain 2
XRCC5	X-ray repair cross-complementing 5
