# Supplementary figures and images for: Integrating Single-Cell, Bulk, and Spatial Transcriptomics Unveils a Novel Ribosome Biogenesis-Related Prognostic Model and Defines RPS19BP1 as a Pro-Oncogenic Regulator in Lung Adenocarcinoma
Source: Int J Mol Sci. 2026 Jun 29;27(13):5864. doi: 10.3390/ijms27135864 (PMC13361457; doi:10.3390/ijms27135864)

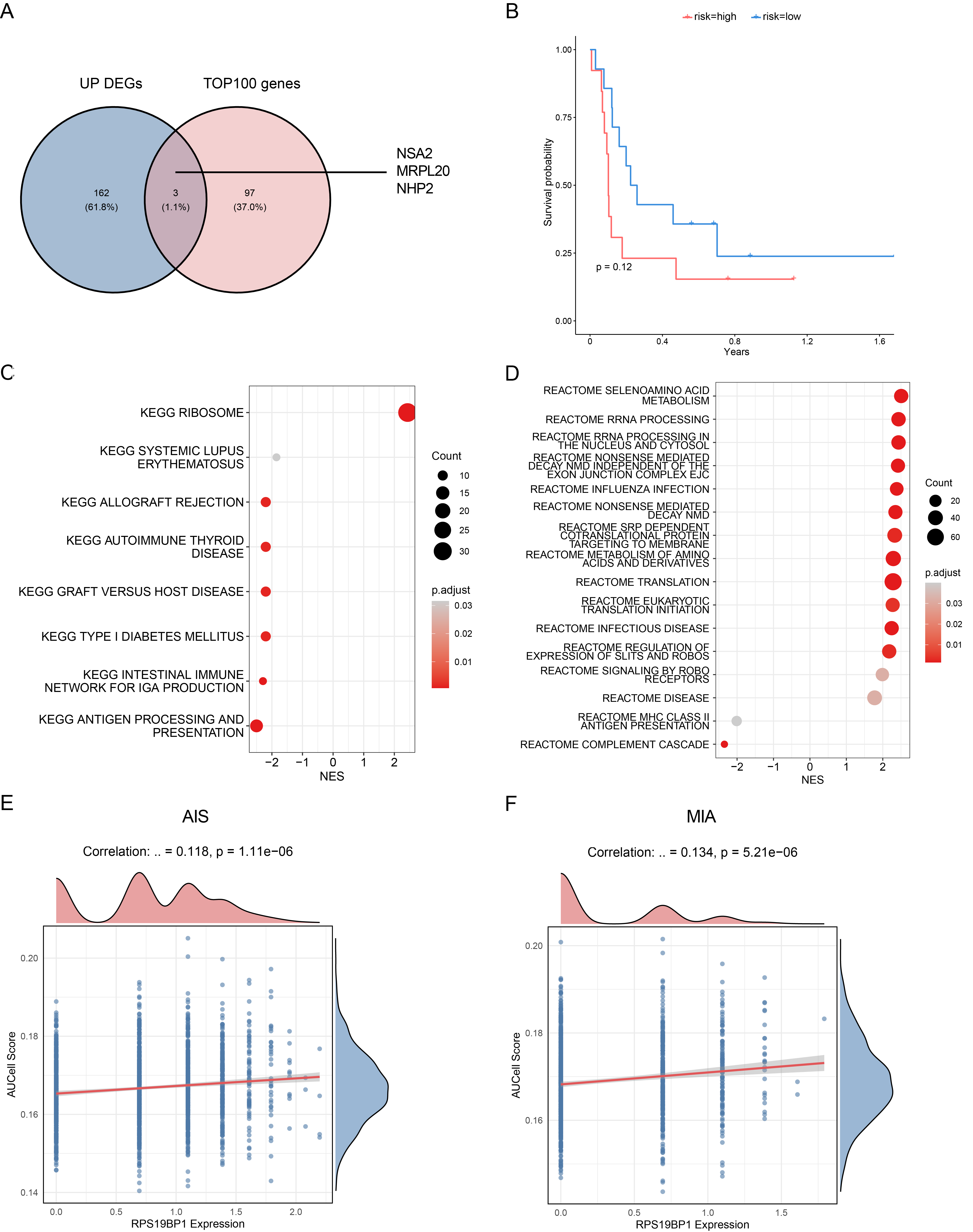

Supplement: Supplementary file 1 [file ijms-27-05864-s001.zip › ijms-4365711-supplementary/Figure S1.png]

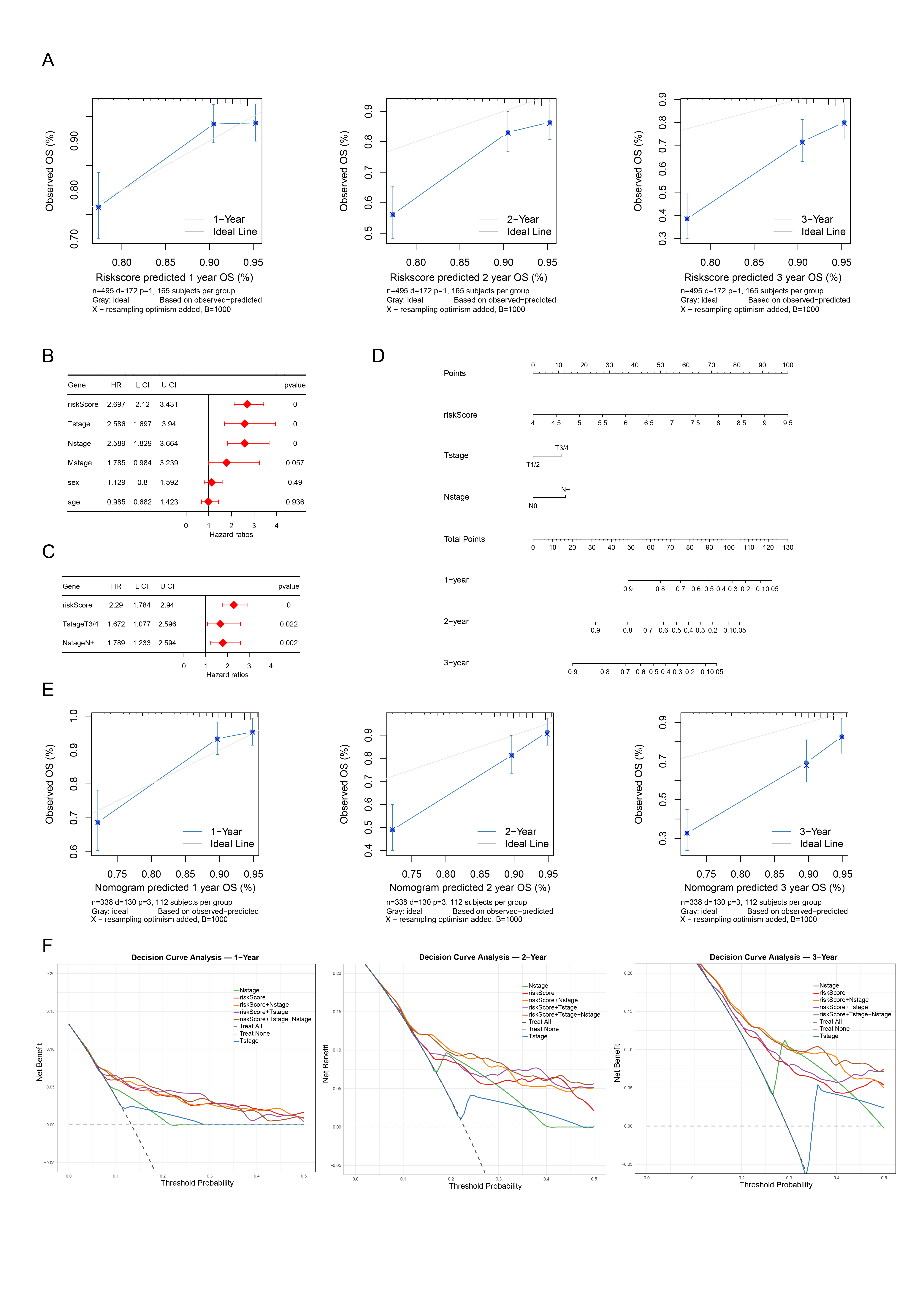

Supplement: Supplementary file 1 [file ijms-27-05864-s001.zip › ijms-4365711-supplementary/Figure S2.png]

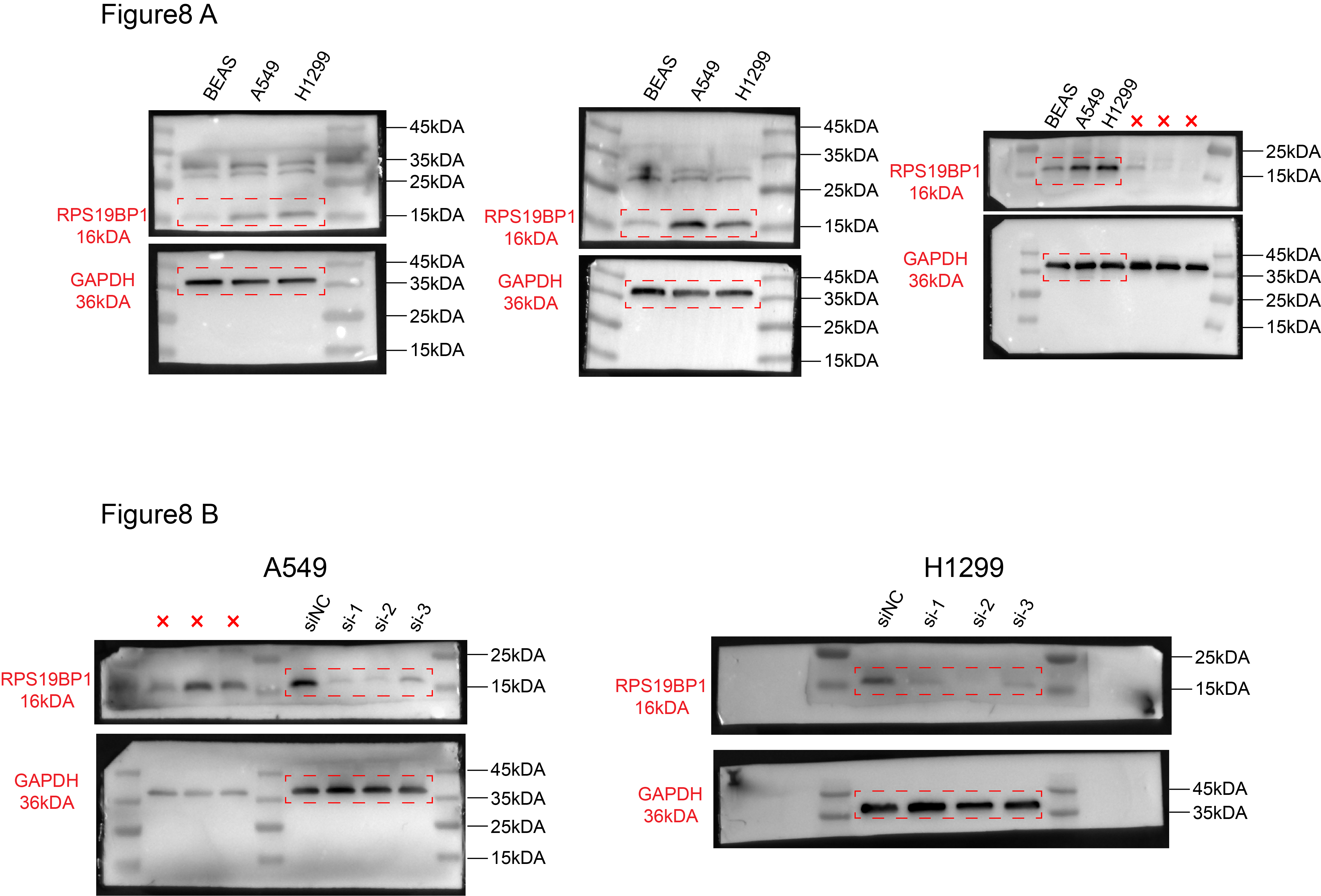

Supplement: Supplementary file 1 [file ijms-27-05864-s001.zip › ijms-4365711-supplementary/Figure S3.png]
